# Experimental and optimization study on the effects of diethyl ether addition to waste plastic oil on diesel engine characteristics

**DOI:** 10.1039/d3ra04489k

**Published:** 2023-08-24

**Authors:** Attasit Wiangkham, Niti Klinkaew, Prasert Aengchuan, Pansa Liplap, Atthaphon Ariyarit, Ekarong Sukjit

**Affiliations:** a Department of Industrial Engineering, Faculty of Engineering, Srinakharinwirot University Ongkharak Nakhon Nayok 26120 Thailand; b Institute of Research and Development, Suranaree University of Technology Muang Nakhon Ratchasima 30000 Thailand; c School of Manufacturing Engineering, Institute of Engineering, Suranaree University of Technology Muang Nakhon Ratchasima 30000 Thailand; d School of Agricultural Engineering, Institute of Engineering, Suranaree University of Technology Muang Nakhon Ratchasima 30000 Thailand; e School of Mechanical Engineering, Institute of Engineering, Suranaree University of Technology Muang Nakhon Ratchasima 30000 Thailand ekarong@sut.ac.th

## Abstract

This study investigates the impact of adding diethyl ether (DEE) to pyrolysis oil derived from mixed plastic waste on engine performance, combustion characteristics, and emissions. The blending of different DEE concentrations (5%, 10%, and 15% by volume) with waste plastic oil (WPO) was analyzed. Experiments were conducted on a four-cylinder diesel engine, varying engine loads while maintaining engine speed. The results indicate that WPO mainly comprises middle-distillate hydrocarbons (52.58% C13–C18 and 26.15% C19–C23). While WPO had lower specific gravity, density, and flash point, it met diesel fuel specifications for kinematic viscosity and cetane index. The addition of DEE led to decreased properties in all blended fuels, except for the cetane index. Engine performance declined with WPO–DEE blends at low engine loads but improved at high engine loads with minimal variation as DEE concentration increased. DEE addition resulted in a shorter ignition delay and earlier combustion, although increasing DEE concentration did not further advance combustion. NO_*x*_ emissions significantly decreased with DEE addition, while HC and CO emissions remained unaffected at high engine loads. To optimize the process, the non-dominated sorting genetic algorithm II (NSGA-II) with generalized regression neural networks (GRNNs) was employed as a surrogate multi-objective function. The GRNNs model demonstrated excellent performance, achieving high *R*^2^ values of 0.952 and 0.918, low RMSE values of 0.659 and 0.310, and MdAPE values of 2.675% and 5.098% for brake thermal efficiency (BTE) and NO_*x*_, respectively. The NSGA-II algorithm with GRNNs model proved successful in predicting the multi-objective function in the optimization process, even with limited data. The Pareto frontier analysis revealed an optimal DEE percentage of approximately 10% to 14% for maximum BTE and minimum NO_*x*_, with engine loads distributed around 30, 40, and 100 N m.

## Abstract 2

The combined NSGA-II algorithm and GRNNs model accurately predicted the multi-objective function, enabling identification of the optimal DEE percentage in WPO and engine operating condition to achieve maximum engine efficiency and minimum emissions.

## Graphical abstract

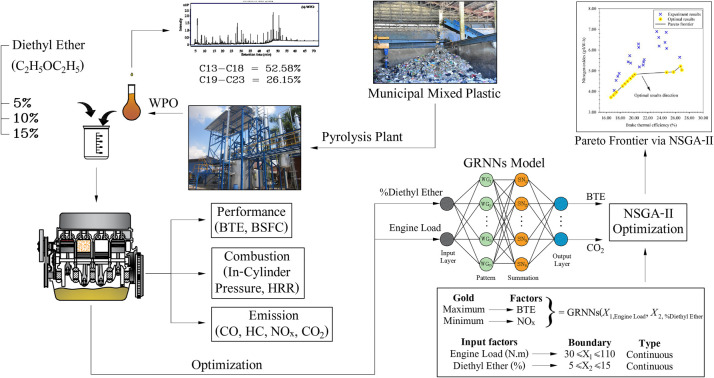

## 1. Introduction

Since the emergence of the COVID-19 virus in 2020 and the implementation of quarantine measures, plastic consumption has significantly increased. The food delivery industry, relying heavily on disposable containers, has been the primary contributor to this surge. Additionally, single-use masks made from polypropylene plastic have become a major source of plastic waste due to their frequent replacement. As a result, plastic waste in Thailand rose by 62% in 2020 compared to the previous year.^1^ The industrial and municipal sectors are the main sources of waste plastic in Thailand.^2^ Among the three primary plastic types, plastic bags are the major contributor, with high density polyethylene (HDPE), low density polyethylene (LDPE), and polypropylene (PP) accounting for 46%, 24%, and 14% of all plastic waste, respectively.

In Thailand, waste disposal is currently managed by burying it in designated areas to minimize air pollution impact. However, the proximity to the equator leads to challenges during the rainy season, causing chemicals, dangerous organic matter, and heavy metals to seep into the soil beneath the buried waste. Similarly, during the summer season, liquid within the buried waste can evaporate and be released into the atmosphere. Proactive waste disposal measures are critical to prevent environmental harm. Despite government efforts to reduce plastic consumption and promote recycling, the quantity of plastic waste continues to surge.^3^ Pyrolysis has emerged as a potential solution to convert plastic waste into fuel oil similar to diesel.^4–7^ This thermal degradation process occurs in an oxygen-free environment at elevated temperatures, producing liquid, gaseous, and solid products, influenced by feedstock composition and pyrolysis parameters.^7^ The fuel properties of waste plastic pyrolysis oils (WPO) vary based on plastic sources,^8–10^ with significant differences observed in kinematic viscosity, flash point, and cetane index compared to diesel fuel. However, the higher density and cetane number of WPO contribute to improved air–fuel mixing, despite its lower flash point. These findings have implications for its potential use as a transportation fuel.

The engine test results revealed that the brake thermal efficiency (BTE) of the waste plastic oil (WPO) was lower than that of diesel fuel, attributed to its high viscosity and poor atomization, resulting in inadequate mixture formation during the premixed combustion phase.^4,5,11^ Conversely, using WPO achieved a higher BTE compared to diesel fuel, primarily due to WPO's higher cetane number, which promoted better air–fuel mixing.^6,12,13^ Prior research indicated that during combustion, WPO generated a lower in-cylinder pressure (ICP) than diesel fuel, attributed to ignition delay. However, the heat release rate (HRR) from WPO was higher than that of diesel fuel, owing to WPO's greater compressibility, leading to a more efficient fuel injection during the combustion process.^6,12^ On the other hand, some scholars argued that both ICP and HRR of WPO were higher than those of diesel fuel.^14^ This could be attributed to WPO's high viscosity and low volatility, resulting in a delay in the preparation of an air–fuel mixture and a sudden surge in cylinder pressure during the premixed combustion phase. Additionally, WPO's higher calorific value and lower atomization during premixed combustion may be responsible for the higher HRR.^4,5,13^ Compression ignition engines are known to emit two primary pollutants: particulate matter (PM) and oxides of nitrogen (NO_*x*_). PM is a hazardous substance that can cause respiratory and dermal problems in the short and long term, as documented in previous research.^15^ NO_*x*_ is a gas identified as a contributor to acid rain.^16^ The generation of PM in engines is associated with the combustion of fuels containing C–C chemical compounds, such as diesel fuel.^17^ Some researchers^6,12^ reported that using WPO as engine fuel resulted in reduced smoke emission, an indicator of PM levels. This reduction was attributed to WPO's high cetane index, leading to a more optimal air–fuel mixture and cleaner combustion. However, other studies reported an increase in smoke emission when using WPO due to its low cetane index.^4,5,11^

Numerous researchers have extensively investigated the utilization of oxygenated fuels, such as biodiesel and ethanol, as potential solutions for reducing PM and NO_*x*_ emissions from diesel engines. While these fuels have demonstrated effectiveness in reducing PM emissions, their use often leads to an increase in NO_*x*_ emissions due to the higher oxygen content, which promotes higher combustion temperatures, favorable for NO_*x*_ formation. This finding has been consistently documented in various studies.^4–6,11–13,17–20^ Several studies have specifically highlighted the benefits of using butanol, an oxygenated fuel with a higher oxygen content than ethanol, in reducing PM emissions when blended with diesel fuel.^21,22^ Additionally, the use of biodiesel blends has been found to result in lower PM emissions compared to regular diesel fuel.^13^ However, when researchers investigated the use of diesel-diethyl ether blends or diethyl ether (DEE) as a supplemental fuel, they found that while PM emissions decreased due to reduced carbon content in the fuel, NO_*x*_ emissions increased due to the higher oxygen content leading to elevated combustion temperatures.^6,19,20,23,24^ Based on the findings reported in previous publications, blending WPO with butanol and DEE resulted in a decrease in BTE and an increase in brake specific fuel consumption (BSFC) primarily due to the lower heating value of butanol and DEE.^4,6^ However, the addition of biodiesel to the blended fuel led to an increase in BTE and a decrease in BSFC, likely attributed to the higher heating value and favorable combustion characteristics of the fuel.^13^ Regarding combustion characteristics, the WPO–DEE blend exhibited lower in-cylinder pressure (ICP) and heat release rate (HRR) compared to diesel fuel due to ignition delay. However, when 5.5% v/v biodiesel was added to the blend, the ICP and HRR approached values closer to those of neat WPO. Moreover, the inclusion of biodiesel in the blend resulted in a significant decrease in unburned hydrocarbon (HC) and carbon monoxide (CO) emissions at low engine load.^6^ DEE is a chemical compound with the molecular formula C_4_H_10_O, and it can be obtained through the dehydration reaction of ethanol. One of its notable properties is an exceptionally high cetane number, which indicates its effectiveness in promoting air–fuel mixing within the combustion chamber, surpassing that of diesel fuel.^6,19,20,23,24^ Thailand's agricultural sector is characterized by extensive cultivation of crops, with a primary focus on those rich in starch content. This abundance of starch-based crops presents an opportunity for ethanol production. Additionally, the sugar production industry in Thailand generates molasses, which can also be harnessed for ethanol production. Consequently, Thailand possesses sustainable resources for DEE production, with all feedstocks originating from the agricultural sector.

Numerous research studies have been dedicated to exploring alternative and renewable fuels derived from agricultural and municipal solid wastes, recognizing their potential for sustainability and their capacity to mitigate greenhouse gas emissions and other pertinent environmental impacts.^25–29^ These investigations have delved into the efficiency and emissions of engines fueled with such alternative fuels and blends, considering various parameters such as fuel type, engine load, compression ratio, fuel injection pressure, and fuel injection timing.^30–32^ The engine's performance has been comprehensively evaluated in terms of BTE, BSFC, and emissions including NO_*x*_, HC, CO, and black smoke, all of which are critical factors in environmental protection. Moreover, these studies have sought to determine the optimal conditions for blended fuels and engine operating parameters, employing optimization algorithms to achieve the highest engine efficiency while minimizing emissions.^33–35^ In the optimization process, the objective function, serving as a surrogate for the relationship between input and output factors in optimization problems, plays a crucial role.^36^ In recent times, artificial intelligence models have emerged as powerful tools for serving as objective functions or surrogate models due to their high accuracy in predicting the behavior of outputs based on problem inputs.^37,38^ Among the widely used algorithms in renewable energy-related problems, the neural network model, inspired by human learning behavior, stands as one of the most commonly employed.^39–41^

The objective of this research is to analyze the impact of blending diethyl ether into waste plastic oil concerning fuel blending ratio and engine operating conditions, with a focus on engine performance, combustion characteristics, and engine-out emissions. Additionally, the study aims to identify the optimal blended diethyl ether ratio and engine operating conditions that achieve both high engine efficiency and minimal emissions. To attain these research objectives, the non-dominated sorting genetic algorithm II (NSGA-II) was employed, a widely-used approach in multi-objective optimization. Furthermore, the study utilized generalized regression neural networks (GRNNs), a type of neural network model known for its suitability in handling small data sets and delivering high prediction performance, to serve as a surrogate for the multi-objective function during the optimization process.^42,43^

## 2. Materials and methods

### 2.1 Test fuels

The waste plastic oil (WPO) utilized in this research was acquired from plastic waste collected within the Suranaree Subdistrict of Nakhon Ratchasima, Thailand. The plastic composition was approximately 20% of the municipal solid waste (MSW), predominantly consisting of polyethylene (PE) and polypropylene (PP). The waste plastic were subjected to composting and separation processes through mechanical biological treatment (MBT) at a pyrolysis facility situated at the Center of Excellence in Biomass, Suranaree University of Technology. The pyrolysis process involved cutting the dried plastic waste into 0.1–0.5 cm aggregates using an agglomerator and feeding it into the pyrolysis chamber using a screw extruder. The pyrolysis chamber was heated to 350–400 °C using a gas burner, while a stirrer installed at the top of the chamber was used to enhance heat transfer to the material. The main pyrolysis products were gases and char, with the latter being periodically removed from the chamber and stored in a separate chamber to cool down before disposal. The gases from the pyrolysis process were flowed into a rectification tower to separate vapor components, with the heavy components being condensed and flowed back to the pyrolysis chamber, and the light components moving upward in the column and condensing into an oil and water mixture. The oil–water mixture was separated by an oil–water separator that employed the difference in liquid density. The resulting oil as called waste plastic pyrolysis oil was pumped to a storage tank and filtered to 1 μm to ensure that it was clean for use in the engine fuel system. More details of the pyrolysis plant used in the study are reported in Arjharn *et al.*^12^ The reference diesel fuel used in this study was commercial diesel purchased from a standard gas station in Thailand, while the analytical grade diethyl ether was purchased from Sigma Aldrich.

The WPO–DEE blended fuel was prepared by mixing using a magnetic stirrer at room temperature for 15 minutes. The DEE-to-WPO blend ratio was varied at 5%, 10% and 15% by volume and named DEE5, DEE10 and DEE15, respectively. All fuel properties and test standards are presented in Table 1. The table illustrates that the specific gravity, density and flash point of the WPO are lower than the standard diesel fuel, while the kinematic viscosity and cetane index are within the range of diesel fuel specifications. In all blended fuels, the properties decreased when DEE was added, as DEE has lower properties compared to diesel fuel. Nevertheless, the cetane index of the blended fuels was higher than that of diesel fuel due to the significantly higher cetane index of DEE. It is observed that the flash point of the resulting WPO is comparatively lower than that of diesel fuel. Moreover, as the concentration of DEE in the blend increases, the flash point of the blend decreases continuously. Given that low flash point fuels are more susceptible to combustion, extra precautions are necessary to prevent accidental ignition and potential fires. To ensure safe handling and usage of WPO blended with DEE, it is imperative to implement appropriate safety measures, including the use of proper storage containers. These measures will contribute to minimizing potential risks associated with the handling of such fuel blends.

**Table 1:** The physical and chemical properties of WPO and its blends

Fuel properties	ASTM test method	Test fuels					
		Diesel	WPO	DEE	DEE5	DEE10	DEE15
Kinematic viscosity@40 °C (cSt)	D445	3.44	3.065	0.240	2.968	2.912	1.992
Specific gravity@15.6 °C	D1298	0.828	0.800	0.716	0.798	0.792	0.790
Density@15.6 °C (kg m^−3^)	D1298	827.19	799.21	714.80	797.21	791.22	789.22
Flash point (°C)	D93	78	36	−40	30	28	24
Cetane index	D976	60.18	68.98	125	69.50	71.46	71.99
Gross calorific value (MJ kg^−1^)	D240	45.39	44.98	36.37	44.48	43.66	43.05

### 2.2 Gas chromatography-mass spectrometry

**Fig. 1:**
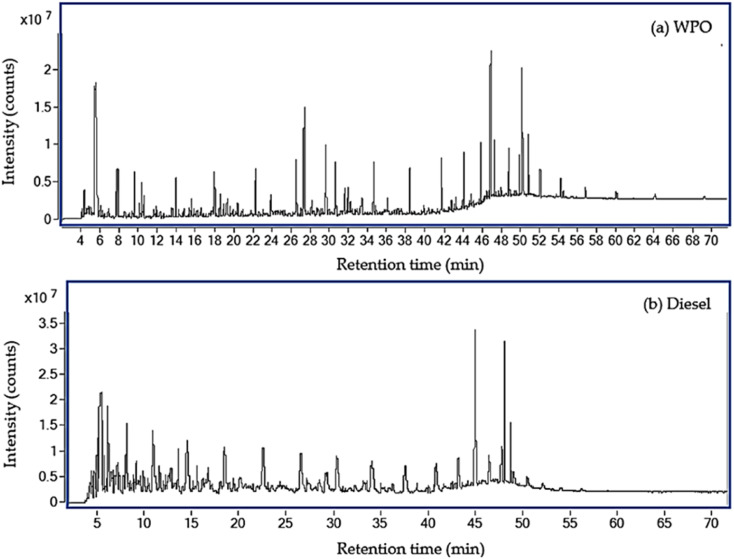
Total ion current chromatogram for WPO and diesel fuel.

**Fig. 2:**
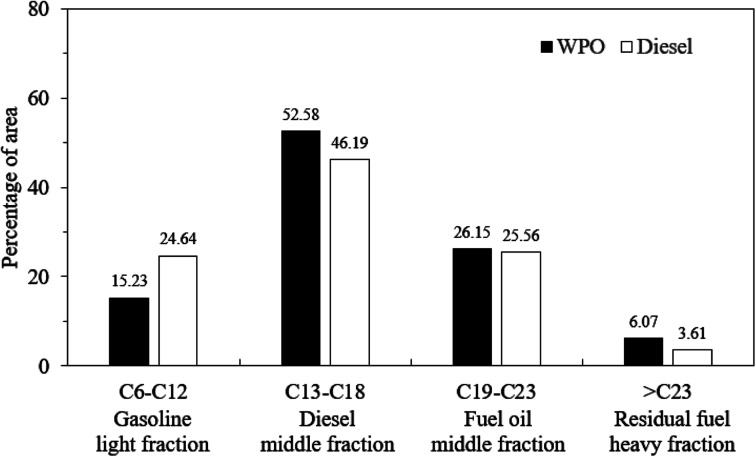
Hydrocarbon compound range of WPO and diesel fuel.

Gas chromatography-mass spectrometry was utilized to identify the chemical components of WPO, while diesel fuel components were identified by GC-MS to serve as the reference fuel. Fig. 1 displays the GC-MS chromatograms of WPO and diesel fuel, respectively, revealing over one hundred peaks for each. The retention times of each peak were compared to standard retention times to identify the components, which are shown in Tables 2 and 3. The analysis identified hydrocarbon substances as the primary components of WPO, ranging from C4 to C29, as well as the presence of aromatic compounds. The study found that WPO consists of hydrocarbons grouped into gasoline (C6–C12, 15.23%), diesel (C13–C18, 52.58%), fuel oil (C19–C23, 26.15%) and residual fuel (>C23, 6.07%). Similarly, diesel fuel consists of hydrocarbons grouped into gasoline (C6–C12, 24.64%), diesel (C13–C18, 46.19%), fuel oil (C19–C23, 25.56%) and residual fuel (>C23, 3.61%). The carbon number distributions of WPO and diesel fuel are illustrated in Fig. 2, which highlights that C6–12 of WPO is lower than diesel fuel, C13–18 of WPO is higher than diesel fuel, and hydrocarbons in a group of fuel oil and residual fuel of WPO are comparable to those in diesel fuel.

**Table 2:** Chemical compound of WPO fuel by GC-MS

Retention time (min)	Chemical compound	Chemical formula	Area (%)
4.31	Pentane, 2,2,4-trimethyl-	C_8_H_18_	0.94
4.39	Heptane	C_7_H_16_	0.33
5.95	Nonane	C_9_H_20_	0.52
7.67	Decane	C_10_H_22_	1.01
7.79	Toluene	C_7_H_8_	2.07
9.59	1-Butanol	C_4_H_10_O	2.28
10.12	Ethylbenzene	C_8_H_10_	0.57
10.35	Undecane	C_11_H_24_	1.76
10.55	Benzene, 1,2-dimethyl-	C_8_H_10_	1.04
11.87	Benzene, 1,4-dimethyl-	C_8_H_10_	0.45
13.50	Benzene, 1-ethyl-4-methyl-	C_9_H_12_	0.54
13.89	Dodecane	C_12_H_26_	2.37
14.20	Mesitylene	C_9_H_12_	0.36
15.56	Benzene, 1,2,3-trimethyl-	C_9_H_12_	0.99
15.83	Dodecane, 4,6-dimethyl-	C_14_H_30_	0.33
16.14	Dodecane, 2-methyl-	C_13_H_28_	0.33
17.97	Tridecane	C_13_H_28_	2.63
22.25	Tetradecane	C_14_H_30_	3.20
26.54	Pentadecane	C_15_H_32_	3.58
27.39	α-Gurjunene	C_15_H_24_	13.76
29.63	β-Gurjunene	C_15_H_24_	5.26
30.64	Hexadecane	C_16_H_34_	3.64
31.57	Alloaromadendrene	C_15_H_24_	1.57
31.96	γ-Gurjunene	C_15_H_24_	1.57
34.60	Heptadecane	C_17_H_36_	3.73
38.40	Octadecane	C_18_H_38_	3.22
41.72	Nonadecane	C_19_H_40_	3.01
43.21	Methyl tetradecanoate	C_15_H_30_O_2_	0.50
44.04	Eicosane	C_20_H_42_	2.72
45.82	Heneicosane	C_21_H_44_	2.54
46.87	Hexadecanoic acid, methyl ester	C_17_H_34_O_2_	9.26
47.29	Docosane	C_22_H_46_	2.27
48.74	Tricosane	C_23_H_48_	1.88
49.91	Methyl stearate	C_19_H_38_O_2_	1.98
50.19	11-Octadecenoic acid, methyl ester	C_19_H_36_O_2_	8.76
50.30	Tetracosane	C_24_H_50_	1.69
50.80	Linoleic acid, methyl ester	C_19_H_34_O_2_	2.99
52.07	Pentacosane	C_25_H_52_	1.33
54.19	Hexacosane	C_26_H_54_	1.07
56.78	Heptacosane	C_27_H_56_	0.81
60.01	Octacosane	C_28_H_58_	0.67
64.08	Nonacosane	C_29_H_60_	0.50

**Table 3:** Chemical compounds of diesel fuel by GC-MS

Retention time (min)	Chemical compound	Chemical formula	Area (%)
4.35	Octane	C_8_H_18_	0.78
4.49	Cyclohexane, 1,4-dimethyl-	C_8_H_16_	0.82
5.05	Nonane	C_9_H_20_	1.67
5.58	Nonane, 4-methyl-	C_10_H_22_	1.61
6.16	Decane	C_10_H_22_	3.62
8.10	Undecane	C_11_H_24_	4.18
9.13	*p*-Xylene	C_8_H_10_	0.94
9.88	*Trans*-2-dodecen-1-ol	C_12_H_24_O	1.09
10.93	Dodecane	C_12_H_26_	5.99
11.62	Benzene, 1-ethyl-3-methyl-	C_9_H_12_	0.92
13.55	Benzene, 1,2,3-trimethyl-	C_9_H_12_	1.62
14.50	Tridecane	C_13_H_28_	6.39
15.56	Benzene, 1,2,4-trimethyl-	C_9_H_12_	1.4
18.49	Tetradecane	C_14_H_30_	6.52
20.11	1-Tetradecene	C_14_H_28_	1.57
22.55	Pentadecane	C_15_H_32_	5.66
26.53	Hexadecane	C_16_H_34_	5.95
30.37	Heptadecane	C_17_H_36_	5.69
34.05	Octadecane	C_18_H_38_	4.3
37.55	Nonadecane	C_19_H_40_	4.01
40.80	Eicosane	C_20_H_42_	3.9
43.18	Heneicosane	C_21_H_44_	3.19
44.96	Hexadecanoic acid, methyl ester	C_17_H_34_O_2_	10.11
46.43	Tricosane	C_23_H_48_	2.36
47.79	Methyl stearate	C_19_H_38_O_2_	2.06
48.07	11-Octadecenoic acid, methyl ester	C_19_H_36_O_2_	7.59
48.68	Linoleic acid, methyl ester	C_19_H_34_O_2_	2.45
49.03	Pentacosane	C_25_H_52_	1.19
50.44	Hexacosane	C_26_H_54_	1.13
52.10	Heptacosane	C_27_H_56_	0.62
53.97	Octacosane	C_28_H_58_	0.34
56.22	Nonacosane	C_29_H_60_	0.33

### 2.3 Engine test

**Fig. 3:**
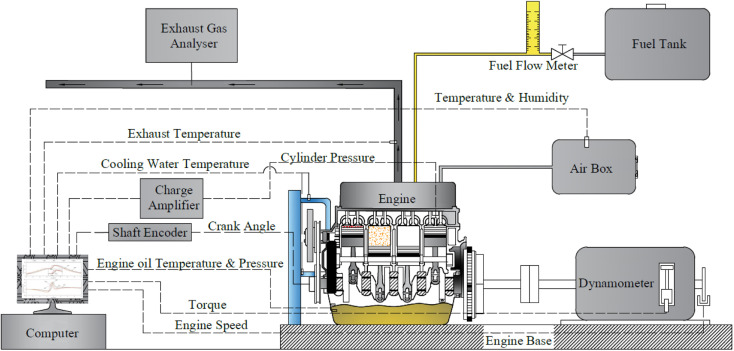
Schematic diagram of the experimental installation.

The study involved experimental investigation on a 4JA1 type, four-stroke, four-cylinder, water-cooled, direct-injection, naturally aspirated diesel engine with a rated power of 64.9 kW@4000 rpm and maximum torque of 171.5 N m@2000 rpm. The engine specifications and experimental schematic diagram are presented in Table 4 and Fig. 3, respectively. A hydraulic engine dynamometer equipped with a load cell was utilized to measure the engine's torque and power output. The engine was operated at a constant speed of 2500 rpm with five different loads, ranging from 30 N m to 110 N m. During the engine tests, diesel fuel was used as the baseline for comparisons with WPO and blended fuels. The air flow rate relative to the engine was measured using an air box, and the volumetric fuel flow rate was measured using a burette and stopwatch. To measure engine crank angle, a Kistler crank angle encoder with a resolution of 0.1 crank angle degree was used. In-cylinder pressure was recorded using a Kistler 6052C piezoelectric pressure transducer coupled with a Kistler 5064C charge amplifier. The in-cylinder pressure data were averaged for 100 cycles at each crank angle for precision enhancement. Exhaust gas emissions were measured using a Testo 350 gas analyzer, with specifications presented in Table 5.

**Table 4:** The technical specifications of the diesel engine

Engine parameters	Specifications
Engine model	4JA1
Engine type	4-Cylinder, 4-cycle, water cooled, direct injection
Bore × stroke	93 mm × 92 mm
Compression ratio	18.4
Displacement	2449 cc
Rated power at 4000 rpm	64.9 kW
Maximum torque at 2000 rpm	171.5 N m
Fuel system	Pump-line-nozzle injection system
Injection nozzle opening pressure	18.1 MPa
Static fuel injection timing	14° bTDC

**Table 5:** Exhaust gas analyzer specifications

Parameter		Measuring techniques	Measuring range	Resolution	Accuracy
Testo 350	NO	Chemiluminescence	0–4000 ppm	1 ppm	±5 < 100 ppm
	NO_2_	Chemiluminescence	0–500 ppm	0.1 ppm	±5 < 100 ppm
	CO	Nondispersive infrared	0–10 000 ppm	1 ppm	±10 < 200 ppm
	HC	Flame ionization detector	0–40 000 ppm	10 ppm	±400 ppm

### 2.4 Optimal solution for engine performance and emission *via* multi-objective optimization

**Fig. 4:**
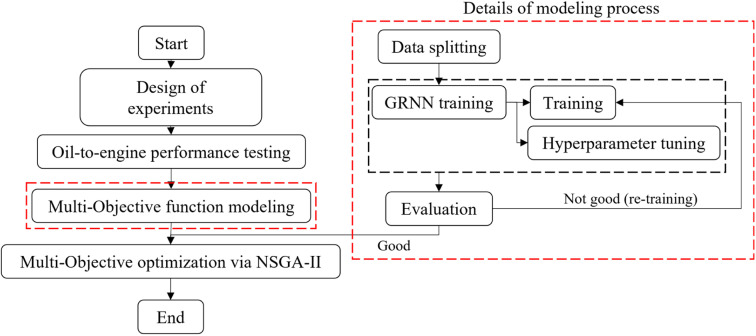
Processing flow chart of the multi-objective optimization of engine and oil conditions.

With regards to the study's objective of investigating the impact of blending diethyl ether with waste plastic oil on engine efficiency and emissions, it is noteworthy that various research efforts have highlighted the influence of the chemical compound ratios mixed into the oil on both engine performance and the resulting emissions. To determine the optimal conditions for oil mixing ratios and usage, a multi-objective optimization technique was employed in this study. The optimization process aimed to address two key metrics: BTE, which serves as an indicator of oil-to-engine performance, and NO_*x*_, a significant emission metric. Typically, these metrics exhibit opposing behaviors, necessitating a comprehensive optimization approach. To assess the factors affecting performance and emissions arising from the oil, namely the engine load (N m) and the percentage of diethyl ether mixed into waste plastic oil, an analysis of variance (ANOVA) was conducted at a significance level of 0.05. The results, presented in Tables 6 and 7, were selected as input factors for the optimization process. Considering the number of objectives to be optimized, the non-dominated sorting genetic algorithm II (NSGA-II), a widely adopted optimization algorithm in the field of energy,^44–46^ was chosen. The flow operation chart depicted in Fig. 4 illustrates the implementation of NSGA-II in this study.

**Table 6:** Analysis of variance of brake thermal efficiency (BTE)

Source	DF	Adj SS	Adj MS	*F*-Value	*P*-Value
Model	14	416.668	29.762	1235.19	0.000
Linear	6	407.799	67.966	2820.77	0.000
Engine load (*A*)	4	406.912	101.728	4221.95	0.000
Percentage of diethyl ether (*B*)	2	0.887	0.444	18.41	0.000
2-Way interactions	8	8.869	1.109	46.01	0.000
*AB*	8	8.869	1.109	46.01	0.000
Error	30	0.723	0.024		
Total	44	417.391			

**Table 7:** Analysis of variance of nitrogen oxides (NO_*x*_)

Source	DF	Adj SS	Adj MS	*F*-Value	*P*-Value
Model	14	31.353	2.239	61.970	0.000
Linear	6	30.659	5.110	141.380	0.000
Engine load (*A*)	4	22.447	5.612	155.270	0.000
Percentage of diethyl ether (*B*)	2	8.212	4.106	113.610	0.000
2-Way interactions	8	0.695	0.087	2.400	0.039
*AB*	8	0.695	0.087	2.400	0.039
Error	30	1.084	0.036		
Total	44	32.438			

#### 2.4.1 Multi-objective function modeling

**Fig. 5:**
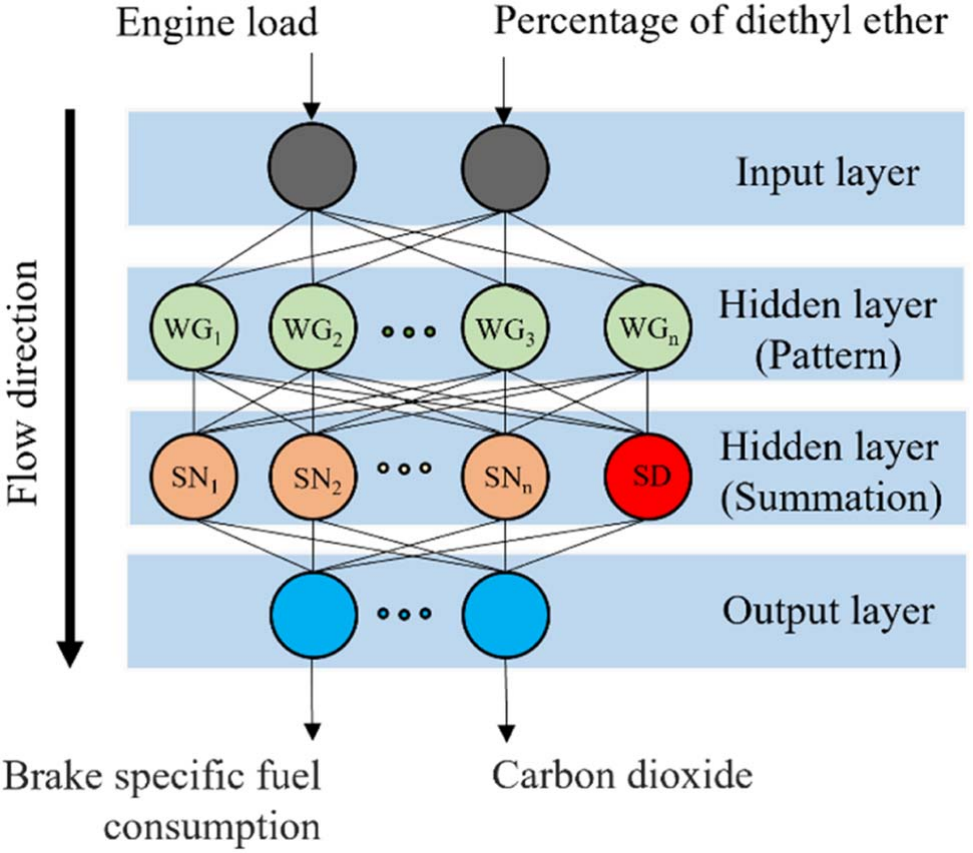
Architecture of generalized regression neural networks modeling.

The initial stage of the multi-objective optimization process involved acquiring the performance and emissions metrics derived from the experimental phase. To establish the relationship between the input factors (engine load and percentage of diethyl ether) and the output factors (comprising various metrics such as BSFC, BTE, NO_*x*_, HC, CO and carbon dioxide (CO_2_)), a surrogate model was constructed using the experimental data mentioned earlier. In this endeavor, the general or generalized regression neural networks (GRNNs) algorithm, known for its suitability in situations involving limited data sets, was chosen.^47–49^ GRNNs, a type of supervised learning neural network, were employed to simulate the problem's behavior, accommodating both linear and nonlinear relationships. The supervised data set, comprising a total of 45 data points, was divided into training and testing sets using the *K*-fold cross-validation technique (with *K* set to 5). Subsequently, the training data set was utilized in the learning phase of the GRNNs model within the MATLAB programming environment. The model architecture, as depicted in Fig. 5, is composed of multiple layers, each with its own functionality, facilitating the representation of the learning process.

The input layer, which constitutes the initial layer of the GRNNs architecture, plays a crucial role in the learning process. Within this layer, the training data set, encompassing both input factors (*X*) and their corresponding output factors (*Y*), is processed. In this layer, the data retains its original format, and the number of neurons is determined by the dimensionality or levels of the input factors under consideration. Hidden layer 1, also known as the pattern layer, constitutes the second layer within the GRNNs architecture. This layer serves the purpose of transforming the input factor data. To accomplish this transformation, a Gaussian kernel function, specifically a radial basis function, is employed. The Gaussian kernel function quantifies the distance between input factors based on the Euclidean distance metric. Mathematically, the Gaussian kernel function can be represented by eqn (1), wherein hyperparameters influence the prediction performance of the GRNNs model.1
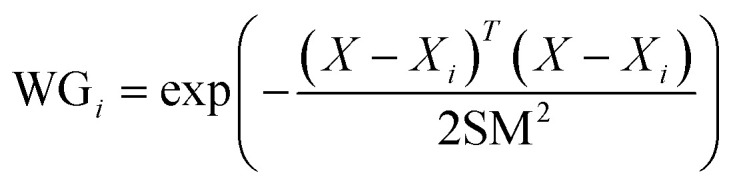
where WG_*i*_ are the transformed input factors (*X*_*i*_) that are known as the weights of the GRNNs model. *X* is the input factors vector that can be written as *X* = [*x*_1_, *x*_2_, *x*_3_, …, *x*_*n*_]^*T*^ while *n* is the number of neurons at the input layer. According to the characteristics of the GRNNs model, the number of neurons in the hidden layer (pattern) cannot be adjusted by the user but is equal to the number of training data that are placed into the model.

Hidden layer 2, referred to as the summation layer, constitutes the third layer within the GRNNs architecture. This layer plays a pivotal role in the GRNNs model, as it utilizes the outcomes obtained from the previous layer for mathematical operations. Within this layer, the neurons can be categorized into two distinct types: numerator neurons (SN) and denominator neurons (SD). The numerator neurons are responsible for summing the results derived from multiplying the transformed input data or weights of the GRNNs from the Pattern layer by the corresponding output data associated with those inputs. This computation can be expressed mathematically through eqn (2). Conversely, the denominator neuron solely performs the summation of the transformed input data or weights of the GRNNs, as depicted by eqn (3). Typically, the summation layer of the GRNNs model consists of one denominator neuron, while the number of numerator neurons is contingent upon the vector of the output factors in the given problem.2
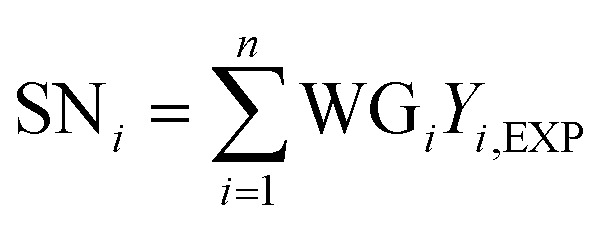
3
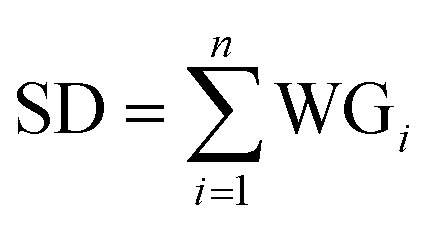


The output layer constitutes the fourth layer within the GRNNs architecture. This layer serves the purpose of transforming the output obtained from the learning process back into the original form of the problem's output. This transformation is accomplished through the relationship between the numerator neurons and the denominator neuron, as expressed by eqn (4).4
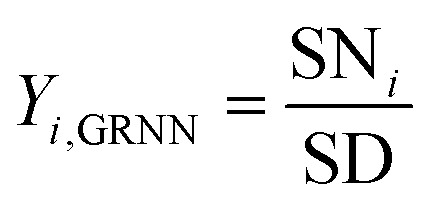


In the process of GRNNs modeling, hyperparameter tuning plays a crucial role in enhancing the prediction performance of the model. Specifically, the focus of hyperparameter tuning in this study was on the smoothing parameter (SM). Generally, the hyperparameter tuning process can be carried out through techniques such as grid search or optimization algorithms. For this study, gradient descent optimization was employed, with the aim of minimizing the average root mean square error. To evaluate the effectiveness of the GRNNs model, which utilized a surrogate model of the multi-objective function, it was imperative to determine whether the model exhibited behavior that was consistent with the actual results of the problem. Performance evaluation was conducted using various regression metrics,^50^ including the coefficient of determination (*R*^2^) as defined in eqn (5), the root mean square error (RMSE) as defined in eqn (6) (which measures scale-dependent accuracy), and the median absolute percentage error (MdAPE) as defined in eqn (7) (which assesses percentage error-based accuracy). These metrics provide valuable insights into the model's performance in replicating the observed behavior and serve as quantitative indicators of its predictive capability.5
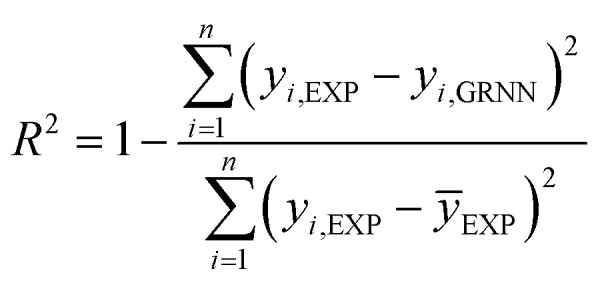
6
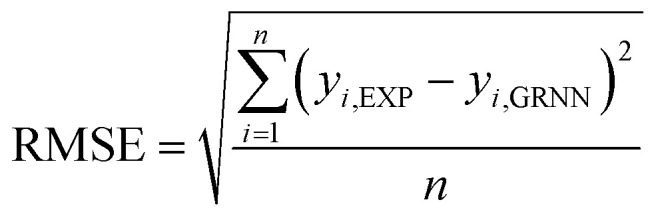
7
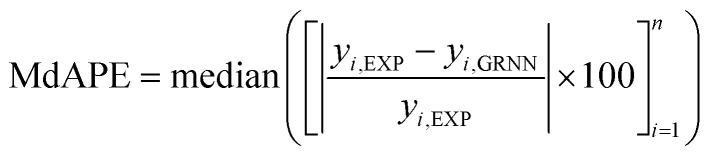
where *y*_*i*,EXP_ are the actual results of output factors in the experiment, *y*_*i*,GRNN_ are the prediction results of the GRNNs model, *n* is the number of observations and *ȳ*_EXP_ is the mean of the actual results of output factors in the experiment.

#### 2.4.2 Multi-objective optimization *via* NSGA-II

Once the GRNNs model, serving as a surrogate for the multi-objective function, was generated and its performance assessed, it was integrated into the multi-objective optimization process. For this particular study, the non-dominated sorting genetic algorithm II (NSGA-II), a widely utilized method for multi-objective optimization problems, was chosen. The NSGA-II optimization was implemented using the MATLAB programming environment, similar to the GRNNs modeling stage. The implementation of NSGA-II aimed to search for the optimal solution that maximizes engine performance while minimizing emissions. The specific details of the NSGA-II implementation can be further elucidated as follows:

(1) The implementation of NSGA-II was started by setting the goals of the optimization process. In this study, according to the characteristics of engine performance and emissions that usually trade-off behavior, the goals were set as maximum brake thermal efficiency, while nitrogen oxides was set as a minimum following eqn (8). To search for the optimal solution, the engine load and percentage of diethyl ether were set with boundaries following the levels used in the experiment, which are shown in eqn (9).8
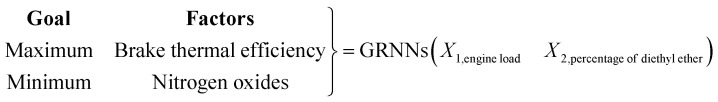
9
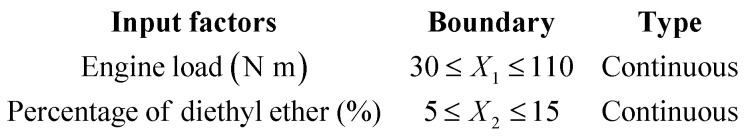


(2) After the goals and boundaries of optimization were set, the initial parameters of the NSGA-II were set. The initial population and number of generations of NSGA-II were set at 100.

(3) After all parameters were set, the multi-objective optimization *via* NSGA-II was started. The prediction results of the GRNNs for each population were generated.

(4) According to the prediction results of the above step, the new parent population were found using crowding testing and non-dominated sorting, and the new offspring population created using crossover and mutation.

(5) Following the setting of the new population, steps (3) and (4) of the optimization process were repeated until the maximum generation was reached.

## 3. Results and discussion

### 3.1 Engine performance

**Fig. 6:**
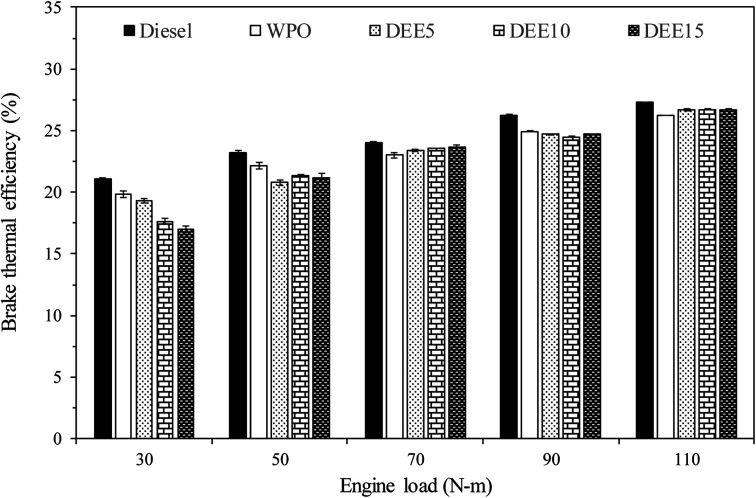
Variation of brake thermal efficiency with engine load.

The results of the load test conducted on the engine are presented in Fig. 6, depicting the various BTE for the test fuels. It is observed that as the engine load increases, BTE also increases for all the test fuels. The increase in BTE with increasing engine load for test fuels can be attributed to improved combustion efficiency, reduced heat losses, and enhanced combustion stability. As the engine load rises, a higher quantity of air–fuel mixture enters the combustion chamber, leading to better mixing and more complete combustion, resulting in higher thermal efficiency. Moreover, at higher engine loads, the relative contribution of heat losses to the cooling system and exhaust decreases compared to useful work output, further improving the overall thermal efficiency of the engine. Additionally, the combustion process becomes more stable due to increased turbulence and better air–fuel mixing, leading to a more consistent and efficient combustion process, further enhancing the overall thermal efficiency.^51^ In comparison to diesel fuel, WPO displays a significantly lower BTE for all engine loads due to its lower gross calorific value, which results in lower heat output compared to diesel fuel for the same amount of fuel used. For the blended fuels, BTE is lower than diesel fuel and WPO, primarily because of the significantly lower gross calorific value of DEE, especially during low-load engine operation where more reduction in BTE is found as the concentration of DEE increases. Additionally, the higher latent heat of vaporization of DEE (360 kJ kg^−1^ (ref. 52)) results in increased heat loss during the preparation of the air–fuel mixture for the pre-mixed combustion process. However, at middle to high engine loads, BTE tends to be slightly higher for all blended fuels compared to WPO, due to the higher cetane index of blended fuels caused by the addition of DEE, which can lead to better air–fuel mixing and provides better pre-mixed combustion. Furthermore, the lower viscosity of blended fuels can enhance fuel atomization and improve the combustion efficiency. The elevated oxygen content and high volatility of DEE also facilitate the combustion rate and enhance the efficiency.^4^ At higher engine loads, the effect of high latent heat of vaporization of DEE can be overcome, as the cylinder temperature is high enough to compensate for heat loss resulting from the energy used in the evaporation of DEE during the air–fuel mixing process. Consequently, the BTE of blended fuels looks stable as the concentration of DEE increases when the engine is operated at high load.

**Fig. 7:**
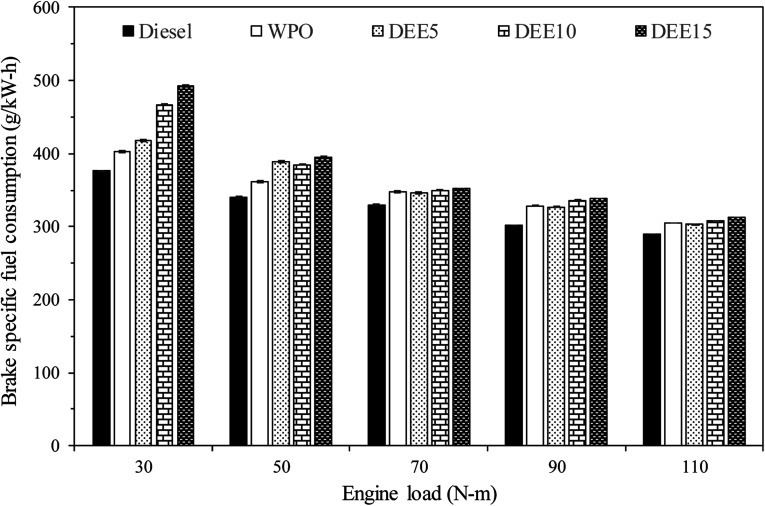
Variation of brake specific fuel consumption with engine load.

The results of the brake specific fuel consumption (BSFC) variation during engine load testing are displayed in Fig. 7. It can be observed that as the engine load increases, the BSFC decreases for all test fuels, which is attributed to more efficient heat conversion and less heat loss. However, when compared to diesel fuel, both WPO and all blended fuels exhibit higher BSFC. This can be explained by the lower gross calorific values of WPO and DEE compared to diesel fuel, which necessitates the engine injecting more fuel to maintain engine load. In addition, at low engine load, blended fuels have significantly higher BSFC than WPO, primarily due to the lower gross calorific value of DEE. However, as the engine load increases, the addition of DEE does not lead to a significant increase in BSFC. This is likely due to the higher cetane index and lower viscosity of blended fuels, which improve fuel atomization and ignition delay, thereby enhancing the combustion process. Additionally, the presence of oxygen in the fuel molecules of DEE can contribute to better combustion.^4,53^ Overall, while the increase in the combustion process due to higher cetane index, lower viscosity and higher oxygen content can balance the decrease in combustion due to higher latent heat of vaporization and lower calorific value of DEE, resulting in similar BSFC when blending more DEE at high engine load.

**Fig. 8:**
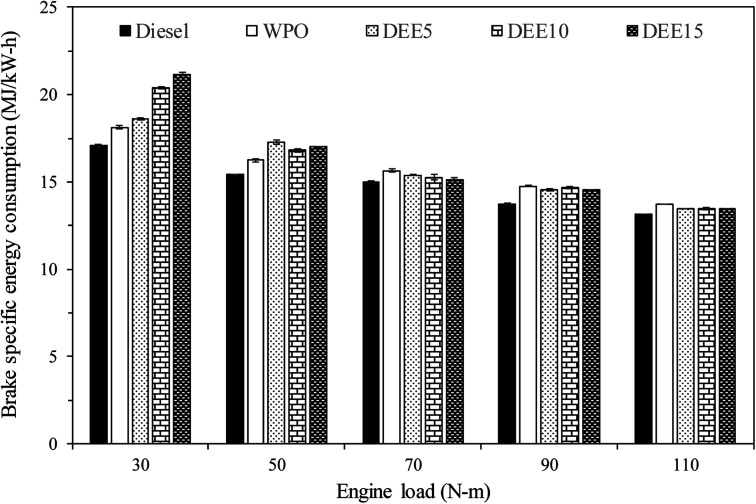
Variation of brake specific energy consumption with engine load.

To provide a comprehensive and precise assessment of the engine's energy efficiency, we evaluated the brake specific energy consumption (BSEC) of the test fuels, as illustrated in Fig. 8. BSEC serves as a measure of the engine's energy consumption rate, taking into consideration both the fuel consumption and energy content. This parameter becomes particularly relevant when comparing the engine's performance under different fuel types with varying energy densities. It is evident from the results that as the engine load increases, the BSEC decreases for all test fuels, indicating a reduced energy consumption rate during engine operation. Notably, the trend in BSEC for all test fuels follows a similar pattern to that observed for BSFC, despite the differing energy densities of the test fuels employed in this study. The combustion of WPO displays a higher BSEC compared to that of diesel fuel. The addition of DEE to the blends does not result in a significant increase in BSEC relative to WPO, except under certain engine load operating conditions where the in-cylinder temperature is insufficient to compensate for the drawback of DEE's high heat vaporization. The rationale used to support the evidence for BSFC across all test fuels can be similarly applied to justify the results of BSEC.

### 3.2 Combustion characteristics

**Fig. 9:**
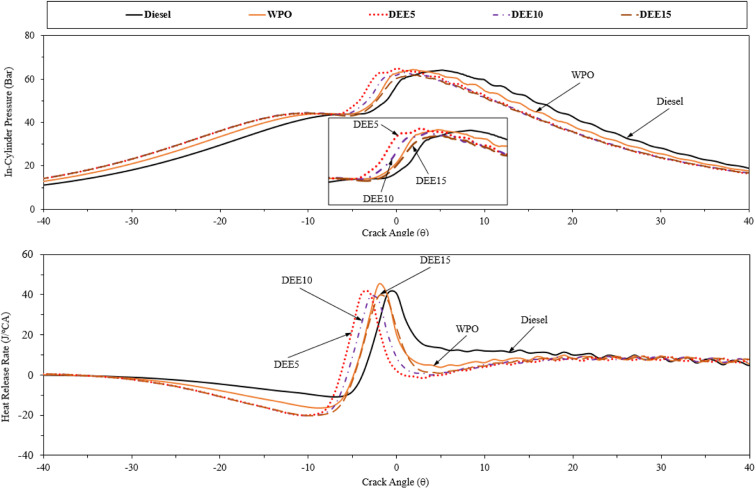
In-cylinder pressure and heat release rate of test fuels under full engine load condition.

Fig. 9 presents the profiles of in-cylinder pressure and heat release rate at full engine load. The in-cylinder pressure distribution over engine crank angle can provide insights into the combustion characteristics of the fuel within the engine cylinder, and the amount of heat released during the combustion process can be determined based on the first law of thermodynamics and the polytropic process assumption.^51^ WPO exhibits earlier combustion with respect to diesel fuel due to its higher cetane index. The addition of DEE to WPO reduces the ignition delay, resulting in an earlier start of combustion. However, increasing the DEE concentration does not result in a significant advancement of the start of combustion due to the more pronounced effect of the reduction in calorific value, and the increase in the latent heat of vaporization.^4^ Moreover, the reduction in density with the addition of DEE, which lowers the bulk modulus of blended fuels, can delay the fuel injection process and contribute to the delay in the start of combustion.^12^ The combustion of blended fuels with 5% and 10% DEE occurs earlier than that of DEE15, while the combustion of DEE15 is closer to that of WPO. Taking into account the start of combustion (SOC), which is defined as the point where the heat release rate experiences a significant rise above the background level, signifying the rapid increase in pressure and temperature within the combustion chamber resulting from the ignition and combustion of the air–fuel mixture, the heat release rate plot indicates that the SOC occurs at 6°, 8°, 9.5°, 9°, and 8.5° before the top dead center for diesel fuel, WPO, DEE5, DEE10, and DEE15, respectively.

### 3.3 Emissions characteristics

**Fig. 10:**
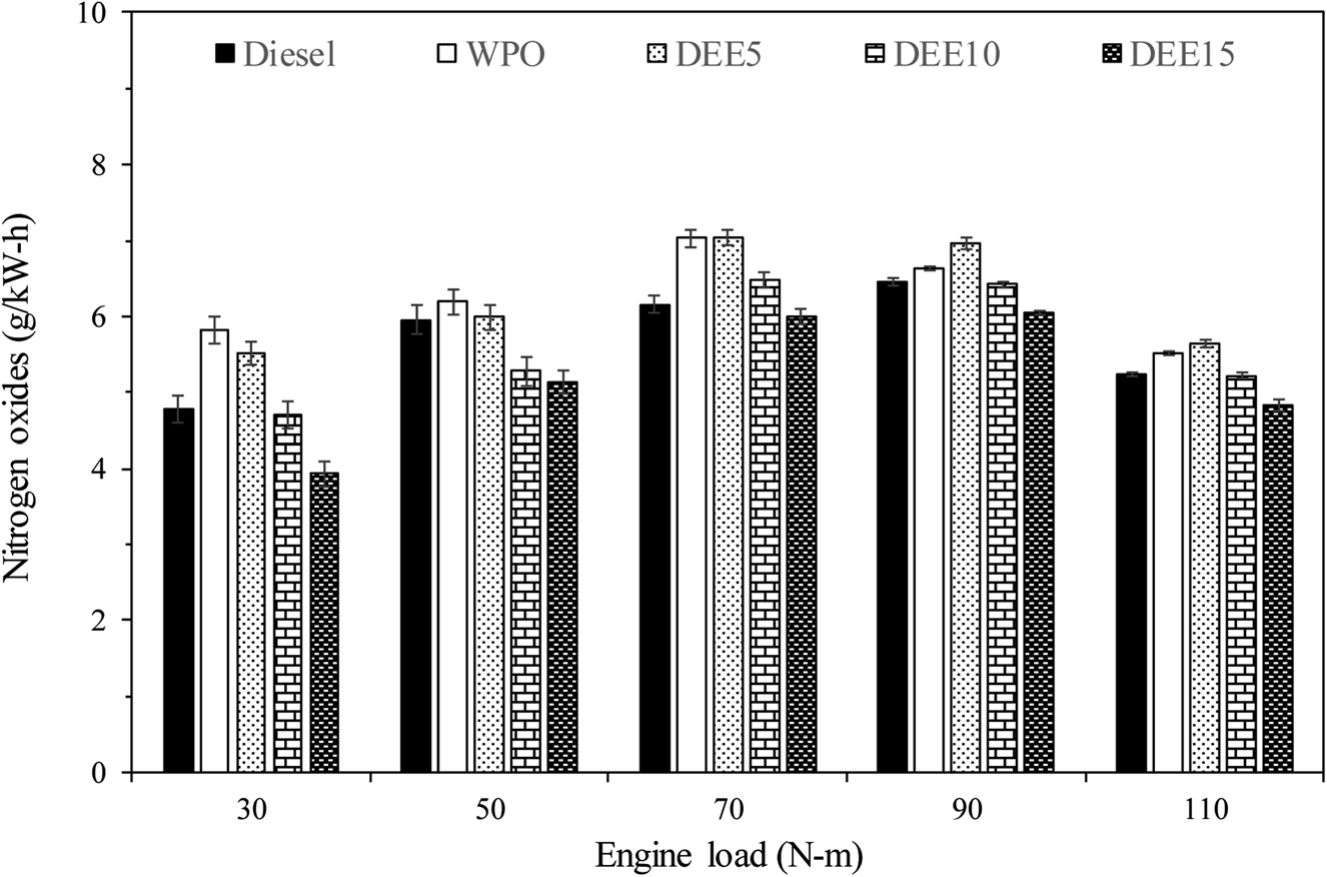
Variation of nitrogen oxides emissions with engine load.

Fig. 10 illustrates the variations in NO_*x*_ emissions with increasing engine loads for all test fuels. NO_*x*_ formation mechanisms mainly include thermal, prompt and fuel mechanisms. The results reveal that WPO produces higher NO_*x*_ emissions than diesel fuel at all engine loads. The higher peak of heat release rate (HRR) observed previously in the HRR section for WPO as compared to diesel fuel may result in a higher maximum in-cylinder temperature during premixed combustion, thereby favoring NO_*x*_ formation. For engine loads of 30, 50 and 70 N m, blended fuels show lower NO_*x*_ emissions than WPO, and the trend continues to decrease as DEE concentration increases, owing to the higher heat of vaporization of DEE, which helps to reduce the in-cylinder temperature.^6^ However, adding 5% DEE does not result in NO_*x*_ reduction at high engine loads (90 and 110 N m), as the small amount of DEE present in the blended fuels does not contribute significantly to NO_*x*_ reduction under these conditions. The effect of high latent heat of vaporization on NO_*x*_ reduction becomes more significant when larger amounts of DEE (10% and 15%) are added, resulting in decreased NO_*x*_ emissions. This trend is also found at engine loads of 90 and 110 N m.

**Fig. 11:**
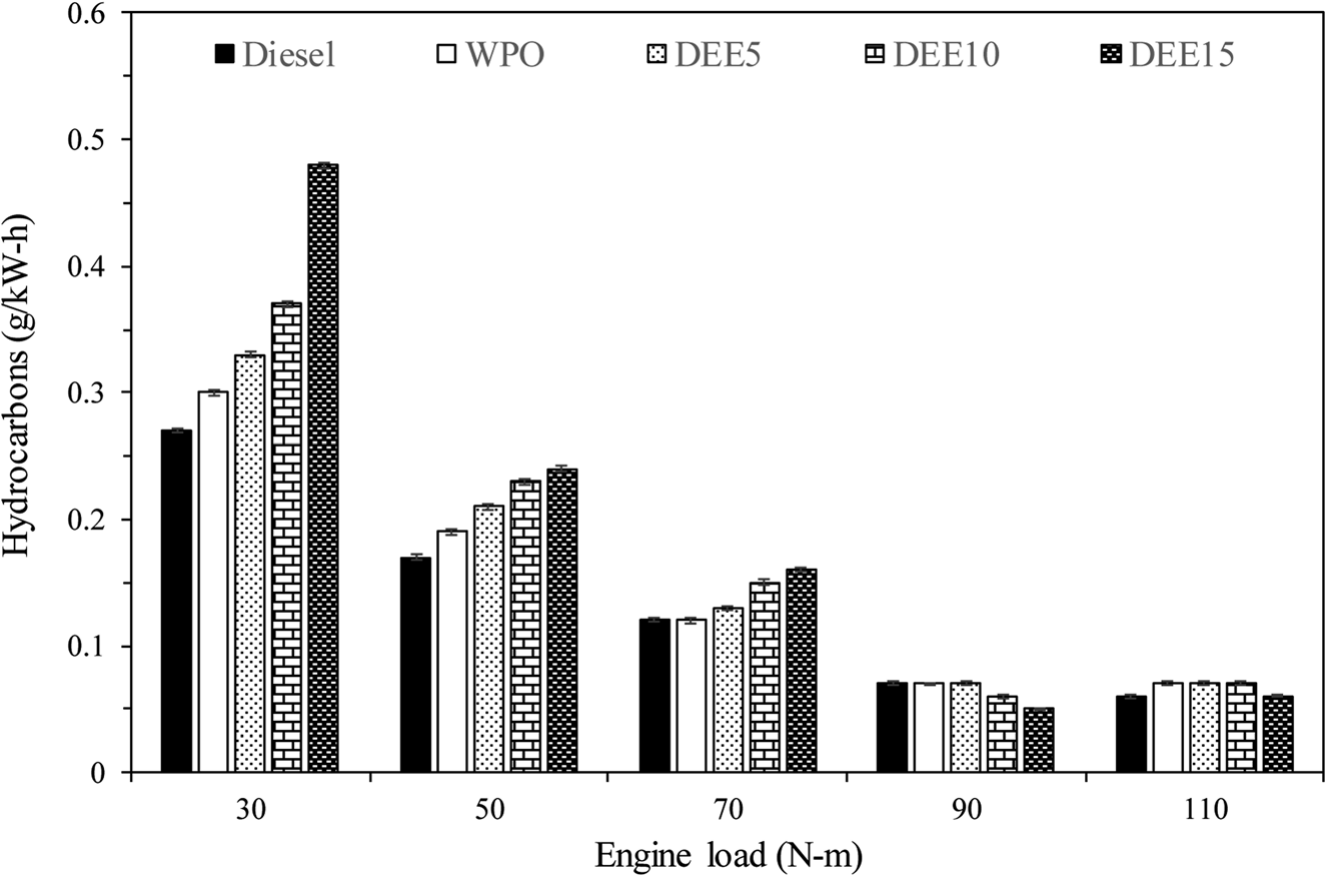
Variation of total hydrocarbon emissions with engine load.

Fig. 11 displays the variations in unburned hydrocarbon (HC) emissions observed with increasing engine loads across all test fuels. The primary causes of HC emissions in diesel engines are cold-region flame-quenching operations and poor combustion. HC emissions of all test fuels generally decrease with increasing engine load due to the higher in-cylinder temperature and improved combustion efficiency. An engine fueled with waste plastic oil and its blended fuels exhibits significantly higher levels than that of diesel fuel at 30 and 50 N m engine load. Additionally, HC emissions tend to increase with an increase in diethyl ether concentration due to the lower in-cylinder temperature resulting from the higher heat of vaporization of DEE. At 70 N m engine load, HC emissions from WPO are similar to those of diesel fuel, while blended fuels continue to exhibit the same trend. At 90 and 110 N m engine loads, HC emissions of WPO and blended fuels are almost identical to diesel fuel, potentially due to a more suitable in-cylinder temperature for improved combustion efficiency. Furthermore, the use of DEE15 at 90 and 110 N m engine load results in lower HC emissions compared to diesel fuel, likely due to the higher availability of oxygen, resulting from the high DEE concentration.^23^ A similar start of combustion between fuels suggests that the oxygen content present in fuel molecules may contribute to improved combustion. These results suggest that the reduction of HC emissions can be achieved by considering high DEE concentration and high engine load, resulting in the benefits of decreasing HC emissions.

**Fig. 12:**
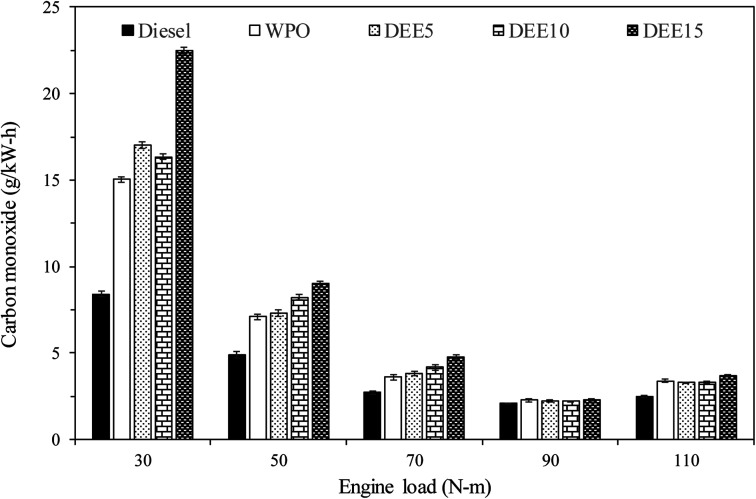
Variation of carbon monoxide emissions with engine load.

Fig. 12 illustrates the variations in CO emissions observed with increasing engine loads across all test fuels. CO emissions are byproducts of incomplete combustion. CO emissions of all test fuels tend to decrease with increasing engine load, due to higher in-cylinder temperature and improved combustion efficiency. Waste plastic oil (WPO) exhibits higher CO emissions than diesel fuel for all engine loads, which may be due to the higher long-chain hydrocarbon content in WPO, requiring a longer combustion period to achieve complete combustion. Additionally, an increase in diethyl ether (DEE) concentration in blended fuels for 30, 50 and 70 N m engine load results in higher CO emissions than diesel fuel, due to lower in-cylinder temperature, resulting in incomplete combustion. The increase in DEE concentration tends to promote higher CO emissions. The higher latent heat of vaporization and lower gross calorific value with increasing DEE concentration can be used to justify such an increase in CO emissions.^6^ However, the difference in CO emissions obtained with the combustion of DEE blends compared to WPO is smaller when the engine is operated at high load (90 and 110 N m), where the in-cylinder temperature is high enough to compensate for the effect of the high latent heat of vaporization of DEE. At high engine load a shorter ignition delay, better fuel atomization and higher availability of oxygen content can contribute to improved combustion under the addition of DEE to WPO, resulting in similar CO emissions between fuel blends and WPO, despite increasing DEE concentration. CO emissions are identical for fuel blends and WPO when the engine is operated at 90 N m.

**Fig. 13:**
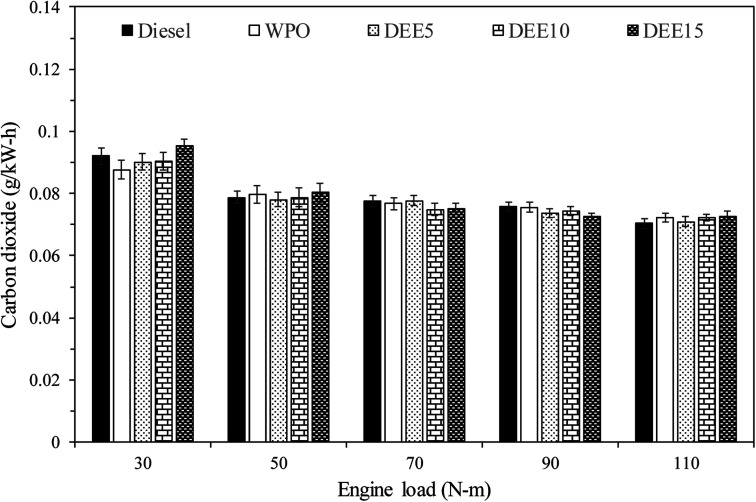
Variation of carbon dioxide emissions with engine load.

Carbon dioxide (CO_2_) is a key indicator of complete hydrocarbon fuel combustion. Fig. 13 displays the variation in CO_2_ emissions with increasing engine loads for all test fuels. CO_2_ emissions per unit of engine power tend to decrease with increasing engine load. The improvement in combustion process with higher BTE can contribute to the reduction of CO_2_ emissions when the engine is operated at higher engine load. It is important to note that an increase in CO_2_ emissions can be observed as engine load increases, when CO_2_ emissions are expressed as a percentage. When compared with diesel fuel, WPO and its blends tend to have lower CO_2_ emissions at low engine load, except for DEE15. However, CO_2_ emissions become similar for all test fuels at high engine load. It should also be noted that DEE blends, with lower calorific value and requiring more fuel to maintain the same engine load, may promote higher CO emissions compared to WPO, even if their contributions to CO_2_ emissions are similar.

### 3.4 Surrogated performance of generalized regression neural networks

**Fig. 14:**
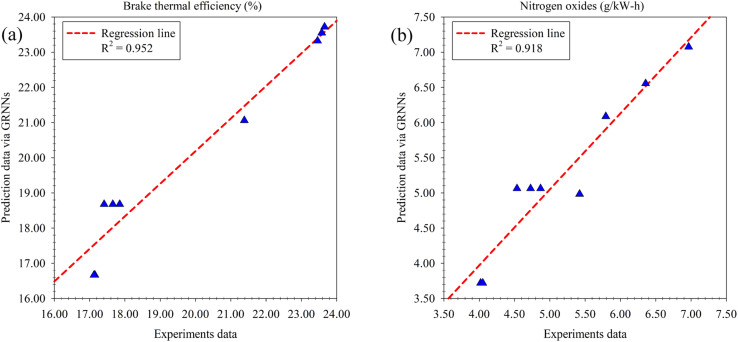
Results of multi-objective function surrogated by GRNNs model (goal of the optimization). (a) Brake thermal efficiency (%). (b) Nitrogen oxides (g kW^−1^ h^−1^).

**Fig. 15:**
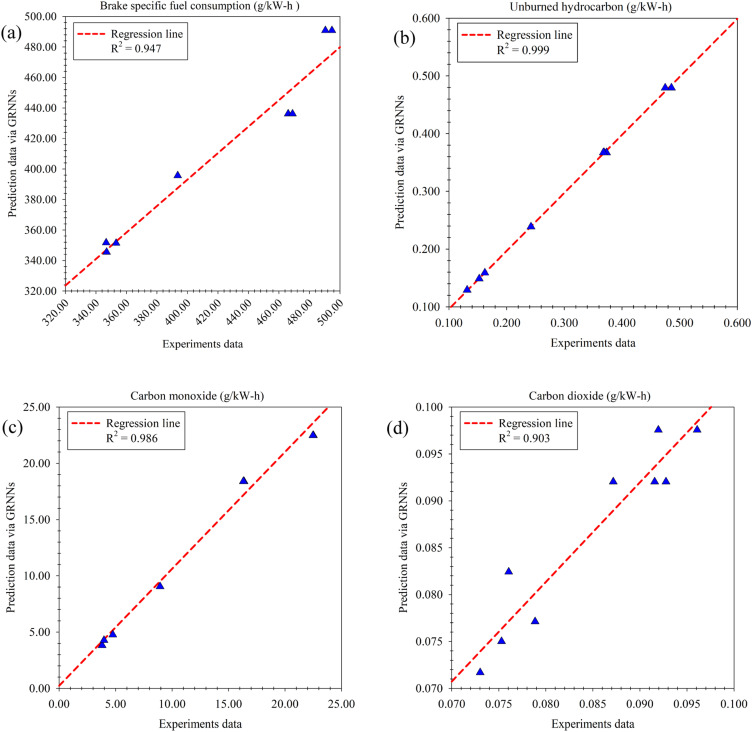
Results of engine performance and emissions surrogated by GRNNs model. (a) Brake specific fuel consumption (g kW^−1^ h^−1^). (b) Unburned hydrocarbon (g kW^−1^ h^−1^). (c) Carbon monoxide (g kW^−1^ h^−1^). (d) Carbon dioxide (g kW^−1^ h^−1^).

In the implementation of multi-objective optimization aimed at finding the optimal solution for engine performance and emissions, a crucial factor influencing the achievement of optimal results is the proximity between the objectives function and the actual experimental results. To address this, a GRNNs model was employed as a surrogate model for the multi-objective function. The performance of this model was thoroughly evaluated using various regression performance metrics, which revealed its high prediction performance. Upon assessing the prediction performance with respect to the optimization goals, namely BTE (%) and NO_*x*_ (g kW^−1^ h^−1^) as illustrated in Fig. 14, it was observed that the GRNNs model exhibited an *R*^2^ value of 0.952 and 0.918 for BTE and NO_*x*_, respectively. These results signify the consistency between the model's predictions and the actual experimental data. Furthermore, the evaluation based on the actual scale of the problem (RMSE) and percentage-based assessment (MdAPE) demonstrated the model's exceptional performance, with an RMSE of 0.659 and 0.310, and an MdAPE of 2.675% and 5.098% for BTE and NO_*x*_, respectively. Notably, the MdAPE values indicate the high prediction performance of the GRNNs model, as they are below the 10% threshold.^54,55^ Additionally, the GRNNs model exhibited impressive predictive capabilities for other engine performance and emission metrics measured during the experimental phase. For instance, when considering BSFC, a key indicator of oil-to-engine performance (Fig. 15(a)), the model achieved an *R*^2^ of 0.947, an RMSE of 5.652 and an MdAPE of 0.181%. Furthermore, for emissions generated from the test fuels, including HC (Fig. 15(b)), CO (Fig. 15(c)) and CO_2_ (Fig. 15(d)), the model demonstrated *R*^2^, RMSE and MdAPE values as follows: (0.999, 0.003, 1.352%), (0.986, 1.184, 1.267%), and (0.903, 0.003, 2.197%), respectively. These findings affirm the strong predictive performance of the GRNNs model across a range of engine performance and emission metrics, further validating its suitability for optimizing engine performance and emissions in the context of the study.

**Fig. 16:**
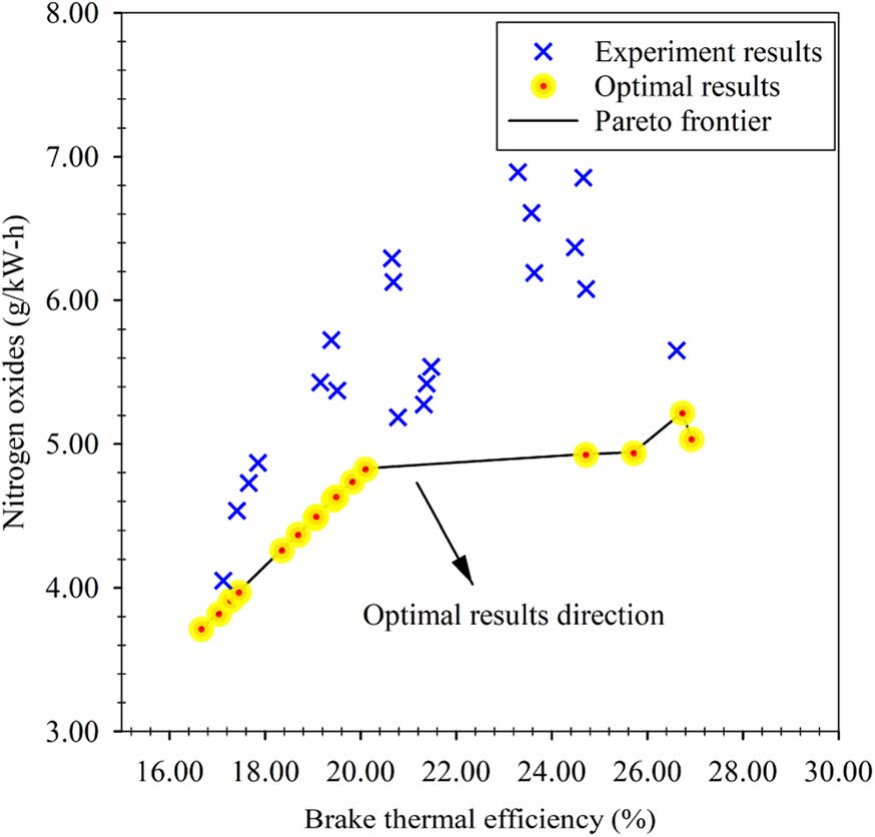
Pareto frontier of multi-objective optimization *via* NSGA-II.

The NSGA-II multi-objective optimization, utilizing the GRNNs model as a surrogate for the multi-objective function, yielded optimal results in the form of a Pareto frontier, as depicted in Fig. 16. The Pareto frontier serves as a boundary delineating the trade-off behavior between oil-to-engine performance and emissions, specifically aiming for maximum BTE and NO_*x*_. The upper region of the frontier line represents suboptimal outcomes, while the lower region denotes the optimal results. Although the evaluation of optimality based on the Pareto frontier graph is relatively straightforward, translating these results into practical implementation poses challenges. The graph solely provides the line of optimal output factors without explicitly indicating the corresponding input factors or solutions. To facilitate the implementation of the results obtained through NSGA-II optimization, the optimal solutions responsible for the optimal outcomes on the Pareto frontier have been presented in both graphical and tabular forms, as illustrated in Table 8. The analysis of the optimal solutions reveals that the optimal percentage of diethyl ether falls within the range of approximately 10% to 14%. On the other hand, the optimal engine load exhibits a more dispersed distribution, spanning around 30, 40 and 100 N m. These findings shed light on the specific input factors that drive the achievement of optimal outcomes in terms of both oil-to-engine performance and emissions, supplementing the information conveyed by the Pareto frontier graph.

**Table 8:** Optimal solution from NSGA-II optimization

Engine load (N m)	Percentage of diethyl ether (%)	Brake thermal efficiency (%)	Nitrogen oxides (g kW^−1^ h^−1^)
38.51	14.17	16.665	3.721
40.03	12.48	20.098	4.833
100.91	12.56	25.711	4.941
42.99	12.47	19.426	4.618
49.99	12.48	18.689	4.376
39.99	10.28	18.351	4.268
41.98	12.42	17.449	3.975
69.19	10.78	19.044	4.491
39.99	12.47	19.486	4.637
100.43	13.13	24.710	4.930
39.99	14.73	17.032	3.826
37.99	11.48	19.825	4.742
39.99	12.04	19.069	4.512
34.98	10.18	17.277	3.919

**Fig. 17:**
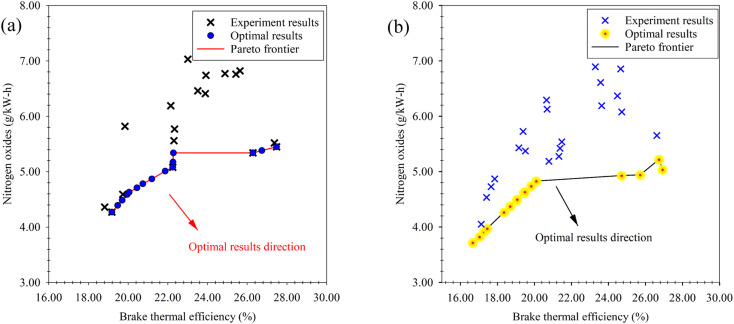
Comparison of the optimal results *via* NSGA-II. (a) Optimal result of waste plastic oil mixed with *n*-butanol. (b) Optimal result of waste plastic oil mixed with diethyl ether.

In our previous study,^44^ which examined the engine performance and emissions resulting from the blending of waste plastic oil produced through the pyrolysis process, with *n*-butanol at the same diethyl ether ratios as used in our present study (5%, 10% and 15%), optimal outcomes in terms of maximum BTE and minimum NO_*x*_ were obtained through the NSGA-II multi-objective optimization. These optimal results are depicted as a Pareto frontier in Fig. 17(a). Upon comparing these optimal results with the findings of our current study (Fig. 17(b)), notable differences emerge. Specifically, with regard to the emissions generated from the blended fuel, it was observed that the addition of diethyl ether yielded more favorable outcomes, characterized by lower emissions. Conversely, when considering engine performance metrics in relation to the blended fuel, *n*-butanol emerged as the optimal solution. These contrasting results suggest that the choice of blending additive for waste plastic oil should be carefully considered, as it entails a trade-off between achieving optimal emissions or optimizing engine performance. The findings from both studies contribute valuable insights for future research and decision-making in the field of fuel additive for improved engine performance and emissions control.

## 4. Conclusions

The study focused on the utilization of pyrolysis oil derived from mixed waste plastic (WPO) and the impact of adding diethyl ether (DEE) at various concentrations (5%, 10%, and 15%, denoted as DEE5, DEE10, and DEE15). The key findings and conclusions are summarized as follows:

• Engine performance decreased with WPO–DEE blends at low engine loads, but significant improvements were observed at high engine loads, with minimal variation as DEE concentration increased.

• Incorporating DEE led to a shorter ignition delay and an earlier start of combustion. However, increasing DEE concentration did not further advance combustion due to higher latent heat of vaporization and lower calorific value, which offset the benefits of DEE's higher cetane index.

• The addition of DEE significantly reduced NO_*x*_ emissions across all engine loads, without detrimental effects on HC and CO emissions at high engine loads (90 and 110 N m).

• The non-dominated sorting genetic algorithm II (NSGA-II) with generalized regression neural networks (GRNNs) effectively served as a surrogate multi-objective function for optimization. The GRNNs model demonstrated high performance, achieving strong predictive accuracy for performance and emission metrics.

• The NSGA-II optimization revealed an optimal DEE percentage of approximately 10% to 14% for maximum BTE and minimum NO_*x*_, with optimal engine loads distributed around 30, 40, and 100 N m.

In order to mitigate the limitations of using waste plastic pyrolysis oils in diesel engines, careful attention must be given to the wax condition in the waste plastic pyrolysis oil, particularly at room temperature. This is crucial to address potential issues that may arise with the fuel injection system. The optimization of relevant parameters in the pyrolysis process for plastic wastes, such as reaction temperature, residence time, catalyst selection, and post-treatment and refining, is essential to minimize the presence of waxes and enhance the overall quality and stability of waste plastic pyrolysis oils. Considering the significance of these endeavors in enhancing the performance and applicability of waste plastic pyrolysis oils in diesel engines, it is recommended that they can be considered as potential areas for future research and development efforts.

## Conflicts of interest

There are no conflicts to declare.

## Supplementary Material
